# *Eucalyptus* Plantation Age and Species Govern Soil Fungal Community Structure and Function Under a Tropical Monsoon Climate in China

**DOI:** 10.3389/ffunb.2021.703467

**Published:** 2021-09-10

**Authors:** Bing Liu, Zhaolei Qu, Yang Ma, Jie Xu, Pei Chen, Hui Sun

**Affiliations:** ^1^Collaborative Innovation Center of Sustainable Forestry in Southern China, College of Forestry, Nanjing Forestry University, Nanjing, China; ^2^Department of Applied Foreign Languages, College of Continuing Education, Nanjing University of Aeronautics and Astronautics, Nanjing, China

**Keywords:** fungal community structure, community function, plantation age, *Eucalyptus* species, tropical monsoon climate

## Abstract

Fungi perform crucial roles in nutrient cycles, but there is limited information on how soil fungal communities vary with stand age and tree species. *Eucalyptus* has been extensively planted in China, which has caused severe soil erosion and water deficiency due to short rotation management. In this study, the fungal community structure and potential function in *Eucalyptus* plantations with different ages (1–5^+^ years) and species (*Eucalyptus urophylla* × *Eucalyptus grandis, Eucalyptus camaldulens*, and *Eucalyptus pellita*) under a tropical monsoon climate in China were characterized by Illumina Miseq coupled with FUNGuild analysis. The results showed that the fungal alpha diversity decreased with an increase in the age of the plantation. Plantations of different ages and species formed distinct fungal communities and potential functional structures, respectively (*p* < 0.05), in which the age of the plantation contributed more to the variations. At high taxonomic levels, the soil fungal community changed from the dominance of orders belonging to Ascomycota (Pleosporales, Chaetothyriales, and Eurotiales) to orders belonging to Basidiomycota (Agaricales, Sebacinales, Cantharellales, and Russulales) with increasing plantation age. The community potential function shifted from the dominance of plant pathogens to a higher abundance of saprotrophs and symbiotrophs. The organic carbon of the soil was the key environmental driver to both the fungal community and potential functional structure. The results provide useful information on the importance of fungi for the management of *Eucalyptus* plantations.

## Introduction

*Eucalyptus*, belonging to the genera of Myrtaceae, is originally from Australia and nearby islands (Grattapaglia et al., 2012). It has been extensively planted on over ~20 million hectares in subtropical and tropical regions because of its fast growth, strong adaptability, and short rotation cycle (Cook et al., 2016). In subtropical and tropical regions of China, farmers have frequently changed their fields to *Eucalyptus* plantations over the past two decades (Yang et al., 2017). Long-term *Eucalyptus* plantations with short rotations and human disturbance have led to a decrease in the quality of soil due to the demand for nutrients, resulting in soil ecological issues, e.g., soil erosion and water deficiency (Laclau et al., 2010; Yang et al., 2017). Furthermore, allelochemicals, such as phenolic acids and terpenoids, are normally released from *Eucalyptus* tissues and accumulate under natural conditions to produce allelopathic effects that can reduce the native biological diversity in *Eucalyptus* plantations (Qin et al., 2018). For instance, the essential oil from *Eucalyptus* can inhibit the proliferation of pathogenic fungi and pupation and feeding of pest insects (Nakahara et al., 2003; Liu et al., 2008; Kaur et al., 2012).

Fungi as decomposers, mutualists, or pathogens (i.e., parasites) in terrestrial ecosystems perform crucial roles in the carbon and nitrogen cycles, which can affect soil quality and plant growth (Peay et al., 2008; Mcguire et al., 2010; Burke et al., 2011). Generally, fungi have stronger effects on the rate of soil organic matter turnover than bacteria because of their higher decomposition ability; as a matter of fact, it is possible to find up to 10^10^ copies of fungal 18S rRNA genes per gram of soil (Ekblad et al., 2013; Lazzaro et al., 2015; Crowther et al., 2019). The relative abundance and occurrence of rare species can shift because of changes in vegetation, soil properties, and forest management (Shade et al., 2014; Uroz et al., 2016). However, because of the complexity of soil and the limitations of identification methods, variations in soil fungal communities and their driving forces remain less understood (Schmidt et al., 2014; Tedersoo et al., 2014; Liu et al., 2015). In recent years, high-throughput DNA sequencing platforms have been proven to be an effective tool for studying environmental microbial communities and how they relate to environmental factors in forests (Schmidt et al., 2014; Uroz et al., 2016).

Vegetation factors, such as stand age and site species composition, can affect fungal communities directly through host–fungi specificity or indirectly through resource input such as root exudates and litterfall (Alguacil et al., 2015; Alfaro et al., 2017). Previous studies have shown that tree species identity is one of the key factors governing soil microbial communities, especially mycorrhizal fungi (Debellis et al., 2006; Horn et al., 2017). For instance, the structure and diversity of fungal communities in temperate and boreal forest ecosystems are strongly dependent on one or few dominant tree species (Sun et al., 2016; van der Linde et al., 2018). Meanwhile, forest stand age can affect fungal community structure and function through changes in the quantity and quality of litter (Schilling et al., 2016). For example, a recent study showed that increasing stand age favored the dominance of soil saprotrophic fungi (e.g., *Mortierellales, Trichoderma*, and *Scheffersomyce*s); however, the results of other research studies showed the opposite trend (Li et al., 2017; Hagenbo et al., 2018). These studies provide useful information for understanding the response of fungal communities to vegetation changes. However, such studies mentioned above are rare in tropical monsoon forests.

*Eucalyptus camaldulens, Eucalyptus pellita*, and *Eucalyptus urophylla* × *Eucalyptus grandis* (female parent *E. urophylla* and male parent *Eucalyptus grandis*), which were selected in this study, and their hybrids are among the nine most planted *Eucalyptus* species, together accounting for more than 90% of *Eucalyptus* plantations (Stanturf et al., 2013). Because of the fact that its adaptability is strong, *E. camaldulens* is the most widely distributed *Eucalyptus* species in the entire Australian continent (Mcdonald et al., 2009). *E. pellita* is naturally distributed in the tropical regions of northeastern Australia, while *E. grandis* usually grows on the edge of rainforests with deep and easy-draining soils on the eastern middle coast (Chippendale, 1981). Unlike most *Eucalyptus, E. urophylla* is one of the few *Eucalyptus* species distributed in the Lesser Sunda Islands of Indonesia outside of Australia, where ecological conditions are similar to those of the east coast of Australia (Pryor et al., 1995). Previous studies on *Eucalyptus* plantations have mainly focused on the effects on soil parameters and mycorrhizal fungal communities, e.g., ectomycorrhiza (ECM) and arbuscular mycorrhiza (Malajczuk et al., 2010; Grbić et al., 2015; Sulzbacher et al., 2018). Recent studies showed that the age and species of *Eucalyptus* plantations significantly affected soil bacterial community, microbial biomass, and enzymatic activities (Qu et al., 2020; Xu et al., 2020). Studies on soil fungal community as a whole in *Eucalyptus* plantations, however, are less common (Castaño et al., 2019). To better understand the impact of *Eucalyptus* planting on soil ecology, it is necessary to assess the dynamic response of soil fungi to *Eucalyptus* plantations. The hypothesis of the authors is that *Eucalyptus* plantations with different ages and species form distinct soil fungal communities. Therefore, we profiled the soil fungal community composition and structure in *Eucalyptus* plantations with different ages and species using high-throughput Illumina Miseq together with FUNGuild. The aims of this study were to (1) investigate how the age of *Eucalyptus* plantations and species impact soil fungal community and functional structures and (2) determine the environmental factors contributing to the variations in fungal communities and functional structures.

## Materials and Methods

### Study Sites

The study sites were located in the nursery base of the China Eucalypt Research Center (CERC, a member of the Chinese Academy of Forestry), northern Leizhou Peninsula, Zhanjiang, southern China (21°27′N, 110°11′E). This region belongs to the northern edge of the Leiqiong district in the humid tropical zone of southern China, and it is situated in the transitional zone between the subtropical humid and marginal tropics zone. The annual average precipitation in the area is ~1,500 mm with an 85.5% rainfall in the rainy season from May to September (Li et al., 2013). The annual average temperature is ~23.1°C, ranging from 17.2°C in January to 38.1°C in July. The soil is orthic ferralsols or brick red based on international or Chinese soil classification, respectively (Liu et al., 2009).

### Sample Collection

In total, six plantations, ~2.2 hectares per plantation, were selected, representing three *Eucalyptus* species [*Eucalyptus urophylla* × *Eucalyptus grandis* (EUG), *Eucalyptus camaldulensis* (EC), and *Eucalyptus pellita* (EP)] with different ages (1, 5, and 11 years old). For EUG and EC, we sampled 1- and 5-year-old plantations; while for EP, we sampled 1- and 11-year-old plantations. The term 5^+^-year-old plantation hereafter refers to the 5-year-old (EUG5y and EC5y) and 11-year-old (EP11y) plantations. The 1-year-old plantations were established from a clear-cut *Eucalyptus robusta* Smith site in early March 2016 and were under similar management with weed control in August 2016 and March 2017 and fertilization with ammonium sulfate in March 2017. The 5^+^-year-old plantations received weed control once a year in August, and the common underground herbs were *Mimosa pudica, Arachis duranensis*, and *Desmodium gangeticum*. The distance between the trees in each plantation was 2 m × 3 m. The depth of soil was ~1 m, and the pH of the soil ranged from 4.3 to 5.4 (Liu et al., 2009). The distance between the 1- and 5^+^-year-old plantations was ~100 m. In each plantation, three subplots (15 m × 15 m) were selected randomly according to the S-shaped route in which three trees were chosen randomly per subplot for sampling. After the removal of litter, three soil cores (100 cm^3^) around each tree with a 1-m distance from the trunk were collected at a depth of 0–10 cm from three directions with 120° and then mixed as one sample, resulting in 9 samples per plantation and 54 samples in total. The samples were put into a carry-on icebox immediately and delivered to the laboratory. All the soil samples were taken on the same day in the rainy season on May 18, 2017. In the laboratory, each soil sample was subdivided into two subsamples after being sieved with a mesh (2 mm) to remove stones, litter, roots, and large particles. One subsample was used to measure the physicochemical properties of the soil, and one was stored at −20°C for DNA isolation until further processing.

### Determination of Soil Physicochemical Properties

Soil water content (SWC) was measured by gravimetry after the soil samples were dried at 105°C for 24 h. The pH of the soil was measured with a digital pH meter in a 1:2.5 soil–water suspension (Kooch et al., 2017). Soil organic carbon (SOC) was measured by the wet oxidation method with potassium dichromate and titration with ferri sulphas (Klute, 1988). The total nitrogen (TN) content of the soil was determined by the semi-micro Kjeldahl method. In addition, the data for the soil microbial biomass, soil fungal biomass (FB), and soil bacterial biomass (BB) in the same study sites were retrieved from Xu et al. (2020), which were used in the analysis of the correlation between community structure and environmental variables. The ratios of soil organic carbon to total nitrogen (C:N ratio) and fungal to bacterial biomass (FB:BB ratio) were calculated.

### DNA Extraction, Amplification of ITS rRNA Region, and Illumina Mise Sequencing

Soil genomic DNA was extracted from 0.3 g fresh soil using the PowerSoil DNA isolation kit (Omega Bio-Tek, Norcross, GA, United States) following the instructions of the manufacturer. The extracted DNA was measured using a Nanodrop-1000 spectrometer (NanoDrop Technologies, Wilmington, DE, United States) and checked by gel electrophoresis. The DNA was subjected to PCR amplification of fungal internal transcribed spacer 1 (ITS1) region using the primer set ITS1F (5′-CTTGGTCATTTAGAGGAAGTAA-3′) and ITS2R (5′-GCTGCGTTCTTCATCGATGC-3′) in a thermocycler 291 PCR system (GeneAmp 9700; Applied Biosystem (ABI), Massachusetts, United States) (Li et al., 2018). PCR reactions were performed in triplicate in a 20-μl mixture containing 4 μl of 5 × FastPfu Buffer (TransGen Biotech, Beijing, China), 2 μl of 2.5 mM deoxyribonucleotide triphosphate (dNTP), 0.8 μl of each primer (5 μM), 0.4 μl of FastPfu Polymerase (ransGen Biotech, Beijing, China), 0.2 μl of bovine serum albumin (BSA), and 10 ng of template DNA. The PCR reactions were conducted using the following program: 3 min of denaturation at 95°C, 36 cycles of 30 s at 95°C, 30 s for annealing at 55°C, and 45 s for elongation at 72°C, and final extension at 72°C for 10 min. The PCR products were checked from a 2% agarose gel and then further purified using the AxyPrep DNA Gel Extraction Kit (Axygen Biosciences, Union City, CA, United States) and quantified using QuantiFluor™-ST (Promega, Madison, WI, United States) according to the protocol of the manufacturer. The purified amplicons were subjected to sequencing with paired-end (PE = 300) on the Illumina MiSeq platform (Illumina, San Diego, CA, United States) according to the standard protocols at Majorbio Bio-Pharm Technology Co. Ltd. (Shanghai, China). The raw reads were deposited into the National Center for Biotechnology Information (NCBI) Sequence Read Archive (SRA) database (accession number: PRJNA580298).

### Sequence Data Processing and Statistical Analysis

The paired-end sequences were processed using the Mothur software (version 1.39.1) following the standard operating procedure (SOP), with the following modifications (Schloss et al., 2009): sequences containing ambiguous (N) bases, homopolymers longer than 8 bp, average quality score lower than 25, putative chimeras (using UCHIME in Mothur), and fewer than 200 bp were omitted (Edgar et al., 2011). Amplification errors were checked using the PCR.seq command. The sequences were pairwise aligned using the align.seq command and pre-clustered with two base-pair differences to remove sequences that were likely due to sequencing errors. The high-quality and unique sequences were classified against the UNITE database (version 8) with a bootstrap cutoff of 80 (Abarenkov et al., 2010). Non-fungal sequences were removed using the remove.lineage command. All the fungal sequences were clustered into operational taxonomic units (OTUs) with 97% similarity using the average neighbor-joining algorithm. All global singletons (OTUs containing only one sequence across all samples) were omitted because of their uncertain origin (Tedersoo et al., 2010). Fungal alpha diversity indexes, such as species richness (Chao 1), diversity (Inverse Simpson's index 1/D), and species evenness (Simpsoneven) were calculated by subsample using the samples with the minimum number of sequences (23,500).

The operational taxonomic units were assigned into functional groups using the FUNGuild database in which the fungi were divided into three basic trophic modes: pathotrophs, symbiotrophs, and saprotrophs (Tedersoo et al., 2014; Nguyen et al., 2016). In addition, the trophic mode can overlap a single fungal taxon. Within these trophic modes, fungi were further divided into a total of 21 functional categories broadly referred to as guilds. For example, the pathotrophs included plant pathogens, animal pathogens, and fungal parasite guilds. The symbiotrophs included arbuscular mycorrhizal fungi, ECM, ericoid mycorrhizal fungi (ErM), and endophyte guilds. The saprotrophs included plant saprotroph, dung saprotroph, litter saprotroph, undefined saprotroph, and wood saprotroph guilds (Nguyen et al., 2016).

All the statistical analyses, except linear discriminant analysis (LDA) coupled with effect size (LEfSe), were performed in the R environment (v3.6.2 or v3.6.3; http://www.r-project.org/). A two-way ANOVA was performed to determine significant differences (*p* < 0.05) in community composition and diversity with plantation age, species, and their interaction as explanatory variables (car, package). One-way ANOVA and Tukey HSD *post-hoc* pairwise comparison tests at *p* < 0.05 were performed (multcomp package) for community diversity. If the data did not conform to a normal distribution, two-tailed Mann–Whitney *U*-tests were performed (spdep and reshape2 packages). Spearman's correlation coefficient and significance were calculated using the package “psych” in R, and the data were scaled by row using the “pheatmap” package. Principle coordination analysis (PCoA) was performed to visualize and detect the difference in fungal community structure or in functional community structure using the “vegan” package with the Bray–Curtis metric (Anderson, 2001). Permutational multivariate analysis of variance (PERMANOVA, adonis in the “vegan” package) was implemented to discern the amount of variation attributed to plantation age, species, and their interaction based on Bray–Curtis distance and 999 permutations. PERMANOVA was also performed to determine the dissimilarity between the community and functional structures of each site. Based on the long length of the first axis of the detrended correspondence analyses (DCA) (i.e., 4.33 for fungi and 4.21 for function), the unimodal ordination method and canonical correspondence analyses (CCAs) were chosen to disentangle relationships between the fungal community or functional structure and environmental variables. The species specialization index (SSI), which is the coefficient of variation in the distribution of fungal performances on host tree species, was computed to quantify the degree of specificity of each fungal species using the ESM package (Poisot et al., 2012). Linear discriminant analysis (LDA) coupled with effect size (LEfSe; http://huttenhower.sph.harvard.edu/galaxy) was performed to identify the fungal or FUNGuild taxa differentially represented among the plantations (Segata et al., 2011). LEfSe was performed with an average relative abundance of taxa of >0.001 in order to hold as many taxa as possible for meaningful comparisons and to remove all rare taxa in the analysis. The criteria for a significance level of fungal taxonomic and functional level were set up with a threshold of an LDA score of >3 and *p* < 0.05.

## Results

### General Analyses of the MiSeq Sequence Data

In total, 1,791,002 reads were obtained after quality control, which were clustered into 3,129 OTUs based on a 97% similarity. Most of the reads (98.69%) could be classified into the phyla of fungi (Supplementary Figure 1). Ascomycota was the most abundant phylum with 49.14% of reads, followed by Basidiomycota (49.09%), Mortierellomycota (0.24%), and Chytridiomycota (0.21%). The order Boletales accounted for 16.13% of the reads, followed by Eurotiales (13.55%), Thelephorales (11.28%), Sebacinales (9.36%), Agaricales (8.19%), and Hypocreales (7.68%). The most abundant genus was *Scleroderma*, a genus of Boletales, which accounted for 11.23% of the reads. Other genera with more than 5% of the reads were *Talaromyces* (7.88%), *Sebacina* (7.22%), and *Thelephora* (5.81%). In addition, 39 OTUs were identified as core OTUs, which existed in more than four soils from each *Eucalyptus* plantation and had an average relative abundance of >0.1% in at least one plantation (Supplementary Figure 2). Of these OTUs, Ascomycota (37 core OTUs, 95.37% of core reads) was more widely distributed than Basidiomycota (2 core OTUs, 4.63% of core reads).

### The Alpha- and Beta-Diversity of Fungal Community

**Figure 1:**
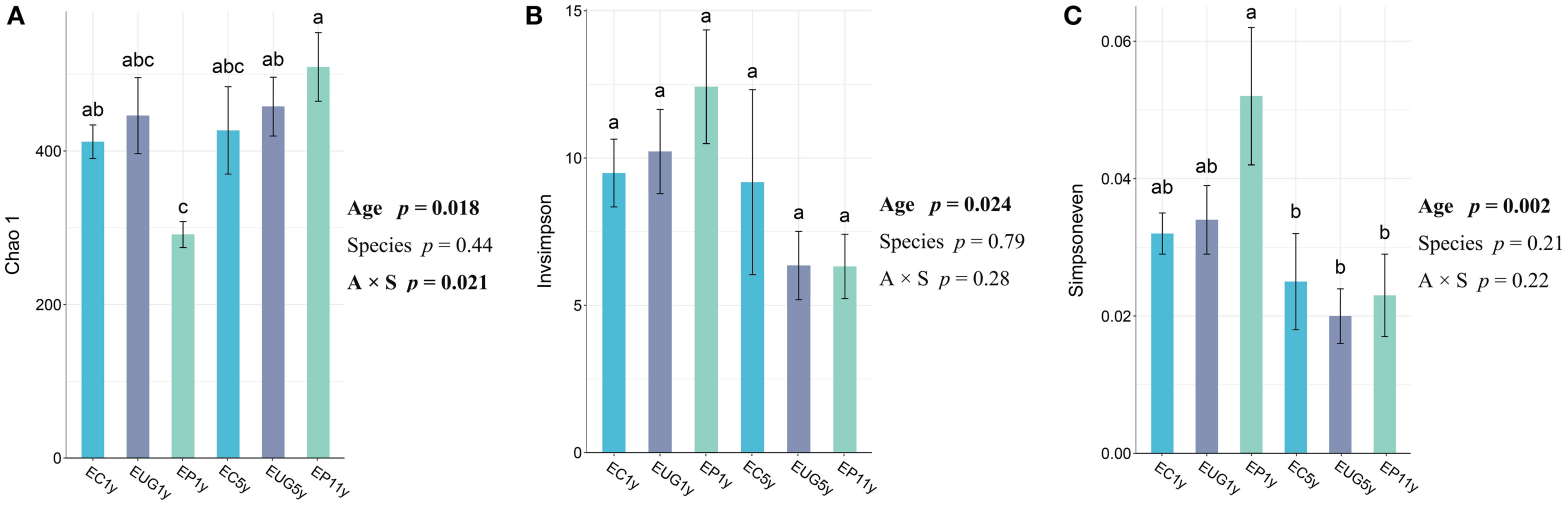
Fungal alpha diversity indexes of **(A)** richness, **(B)** diversity, and **(C)** evenness in *Eucalyptus* plantations with different ages and species. Two-way ANOVA model summary for responses of alpha diversity indexes and explanatory variables of *Eucalyptus* plantation age, species, and their interaction. Explanatory variables deemed statistically significant are bolded. One-way ANOVA and Tukey [honestly significant difference (HSD) *post-hoc* pairwise comparison tests (*p* < 0.05) were performed after checking for assumptions of normality and homoscedasticity. Accessions with the same letter were not significantly different. EC, *Eucalyptus camaldulensis*; EP, *Eucalyptus pellita*; EUG, *Eucalyptus urophylla* × *Eucalyptus grandis*; 1y, 1 year; 5y, 5 years; 11y, 11 years.

All fungal alpha diversity indices (richness, diversity, and evenness) of the 1-year-old plantations differed from that of the 5^+^-year-old plantations (Figure 1). The 5^+^-year-old plantations had significantly higher richness than the 1-year-old plantations (*p* = 0.018). In contrast, the 1-year-old plantations had a higher diversity and evenness than the 5^+^-year-old plantations (*p* = 0.024 and *p* = 0.002, respectively). In addition, the fungal richness in the EP 1-year-old plantation differed from that in the EC 1-year-old plantation, but no significant difference was observed for fungal richness, diversity, and evenness among the *Eucalyptus* species. However, the interaction between plantation age and species showed a significant effect on fungal richness (*p* = 0.021). The fungal diversity showed a positive correlation with evenness (Supplementary Table 1; *R* = 0.9184, *p* < 0.001).

**Figure 2:**
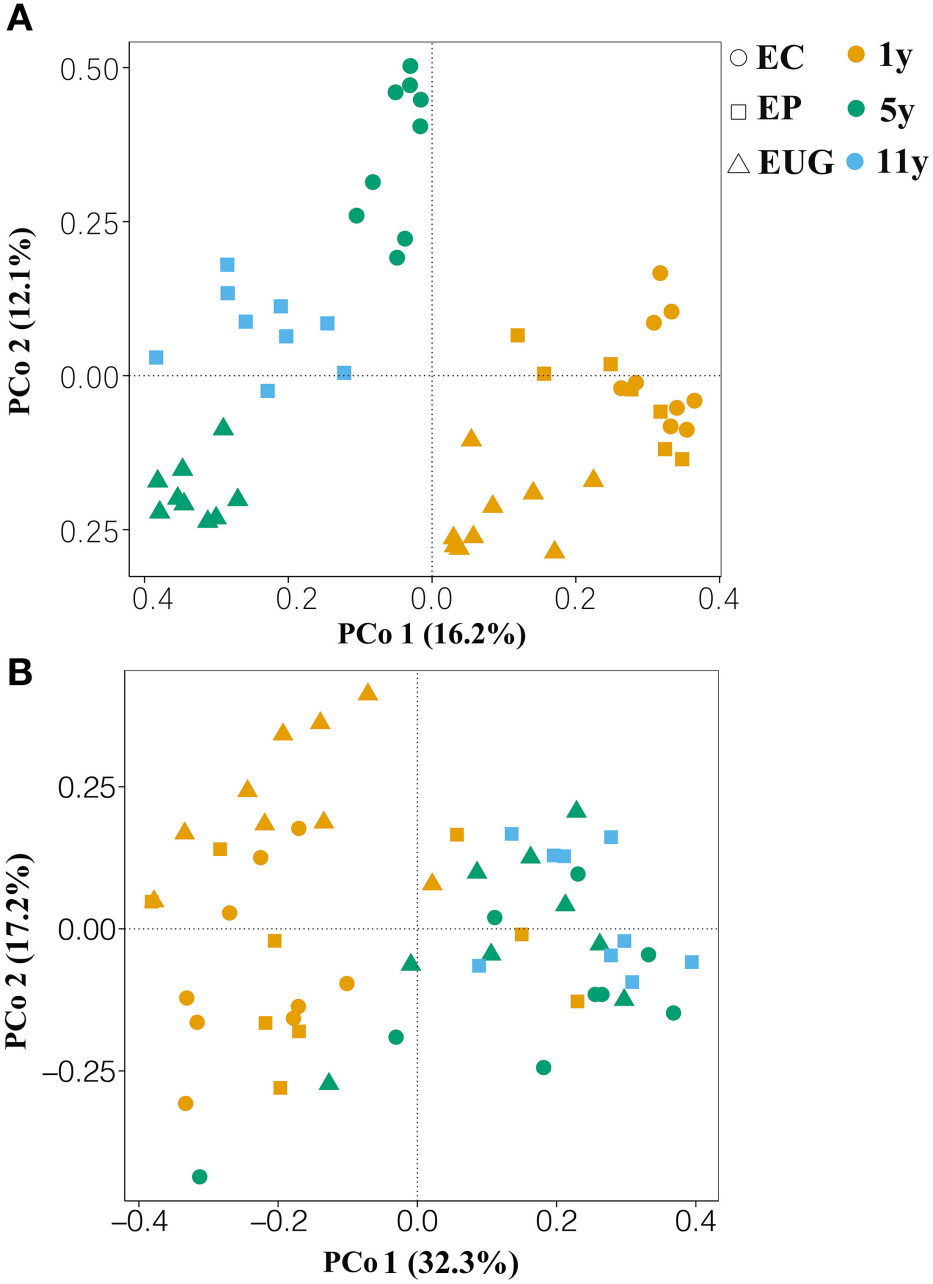
Principle coordination analysis (PCoA) based on Bray–Curtis similarity matrices showing the **(A)** fungal and **(B)** functional community structures in different soils. EC, *E. camaldulensis*; EP, *E. pellita*; EUG, *E. urophylla* × *E. grandis*; 1y, 1 year; 5y, 5 years; 11y, 11 years.

The principle coordination analysis showed that the six plantations formed five distinct fungal communities (Figure 2A; PERMANOVA, pseudo-F > 4.89, *R*^2^ >0.23, *p* < 0.01). The EP1y- and EC1y-plantations did not separate by fungal community (PERMANOVA, pseudo-F = 1.35, *R*^2^ = 0.08, *p* >0.05). Correspondingly, the 1- and 5^+^-year-old plantations harbored distinct fungal compositions and confirmed a clear separation along PCo 1 (explaining 16.2% of the overall variation), whereas PCo 2 explained 12.1% of the overall variation and separated mainly the *Eucalyptus* species. Moreover, plantation age accounted for a larger proportion of variation (PERMANOVA, pseudo-F = 7.09, *R*^2^ = 0.22, *p* < 0.001) in fungal community structure compared with the species (PERMANOVA, pseudo-F = 4.74, *R*^2^ = 0.16, *p* < 0.001).

### Fungal Taxa Differences in *Eucalyptus* Plantations

**Figure 3:**
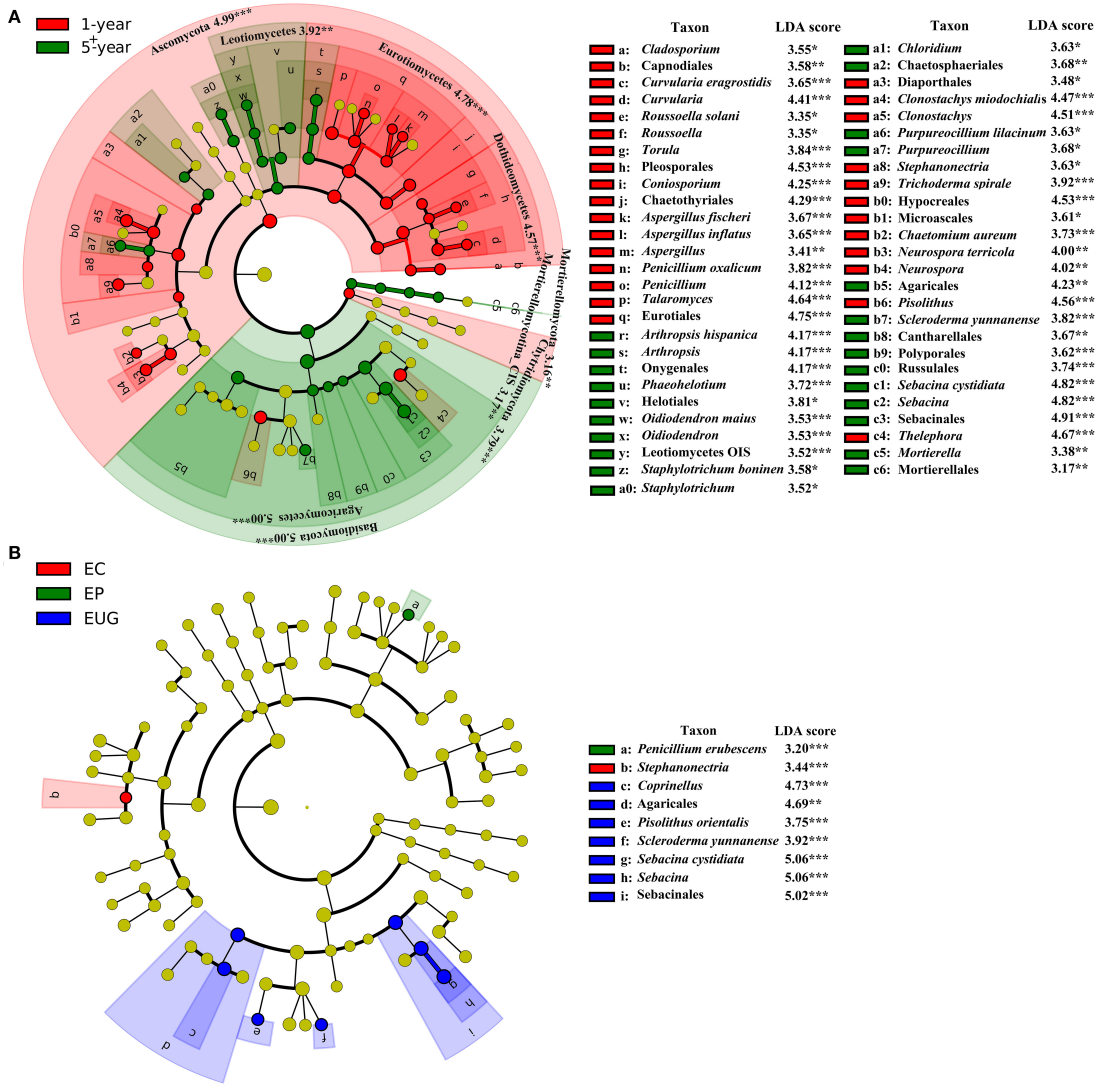
Fungal taxa (without family) significantly differentiated in *Eucalyptus* plantations with **(A)** different ages and **(B)** different species, identified by linear discriminant analysis coupled with effect size (LEfSe) using parameters of a threshold of linear discriminant analysis (LDA) score > 3 and *p* < 0.05. From the inner circle to the outer circle of tree diagrams are fungal taxa of phylum, class, order, genus, and species. The red and green nodes and shades shown in **(A)** represent fungal taxa that play an important role in the 1- and 5^+^-year-old plantations, respectively; the red, green, and blue nodes and shades shown in **(B)** represent fungal taxa that play an important role in EC, EP, and EUG plantations, respectively; the yellow nodes represent fungal taxa that do not play an important role in each treatment. EC, *E. camaldulensis*; EP, *E. pellita*; EUG, *E. urophylla* × *E. grandis*; CIS, Class *Incertae sedis*; OIS, Order *Incertae sedis*. *signifiant level of 5%; **significant level of 1%, and ***significant level of 0.1%.

At higher taxonomic levels, Ascomycota (the phylum and its orders Pleosporales, Chaetothyriales, and Eurotiales) and Chytridiomycota had a higher abundance in the 1-year-old plantations, whereas there was a distinct increase in the relative abundance of Basidiomycota (the phylum and its orders Agaricales, Sebacinales, Cantharellales, and Russulales), Mortierellomycota (the phylum and its order Mortierellales), and ascomycetes Helotiales in the 5^+^-year-old plantations (Figure 3A, *p* < 0.01). At lower taxonomic levels, the genera *Curvularia, Coniosporium, Aspergillus, Penicillium, Talaromyces*, and *Clonostachys* were more abundant in the 1-year-old plantations, whereas *Arthropsis, Purpureocillium, Sebacina*, and *Mortierella* were more abundant in the 5^+^-year-old plantations (*p* < 0.05). The other taxa (unique to a specific site) with significant differences in abundance between plantations are listed in Figure 3A.

Only a small number of taxa differed significantly among the *Eucalyptus* species. The order Agaricales and its genus *Coprinellus*, Sebacinales, and its genus, *Sebacina* and *Pisolithusorientalis*, were more abundant in the EUG plantations, whereas the genus *Stephanonectria* was more abundant in the EC plantations (Figure 3B; *p* < 0.01). Sebacinales was the most specialized order with the highest mean SSI (Supplementary Figure 3A). In addition, Agaricales and its genus *Coprinellus* had a higher mean SSI (Supplementary Figure 3).

### Fungal Community Structure of Functional Guilds

For the functional groups, a total of 1,060,621 reads (59.22% of the total reads) were assigned to the three functional guilds (Supplementary Figure 4). The obligate saprotrophs were the most dominant functional guilds, covering 39.34% of the assigned reads, followed by obligate symbiotrophs (27.1% of the assigned reads), obligate pathotrophs (14.74%), pathotrophs–saprotrophs–symbiotrophs (12.13%), pathotrophs–saprotrophs (4.5%), pathotrophs–symbiotrophs (1.32%), and saprotrophs–symbiotrophs (0.87%) (Supplementary Figure 4). Similar to the fungal community structure, the PCoA showed that the six plantations formed five distinct functional structures (Figure 2B; PERMANOVA, pseudo-F = 2.06, *R*^2^ >0.12, *p* < 0.05). The EP1y- and EC1y-plantations did not differ in functional structure (PERMANOVA, pseudo-F = 1.54, *R*^2^ = 0.09, *p* >0.05).

**Figure 4:**
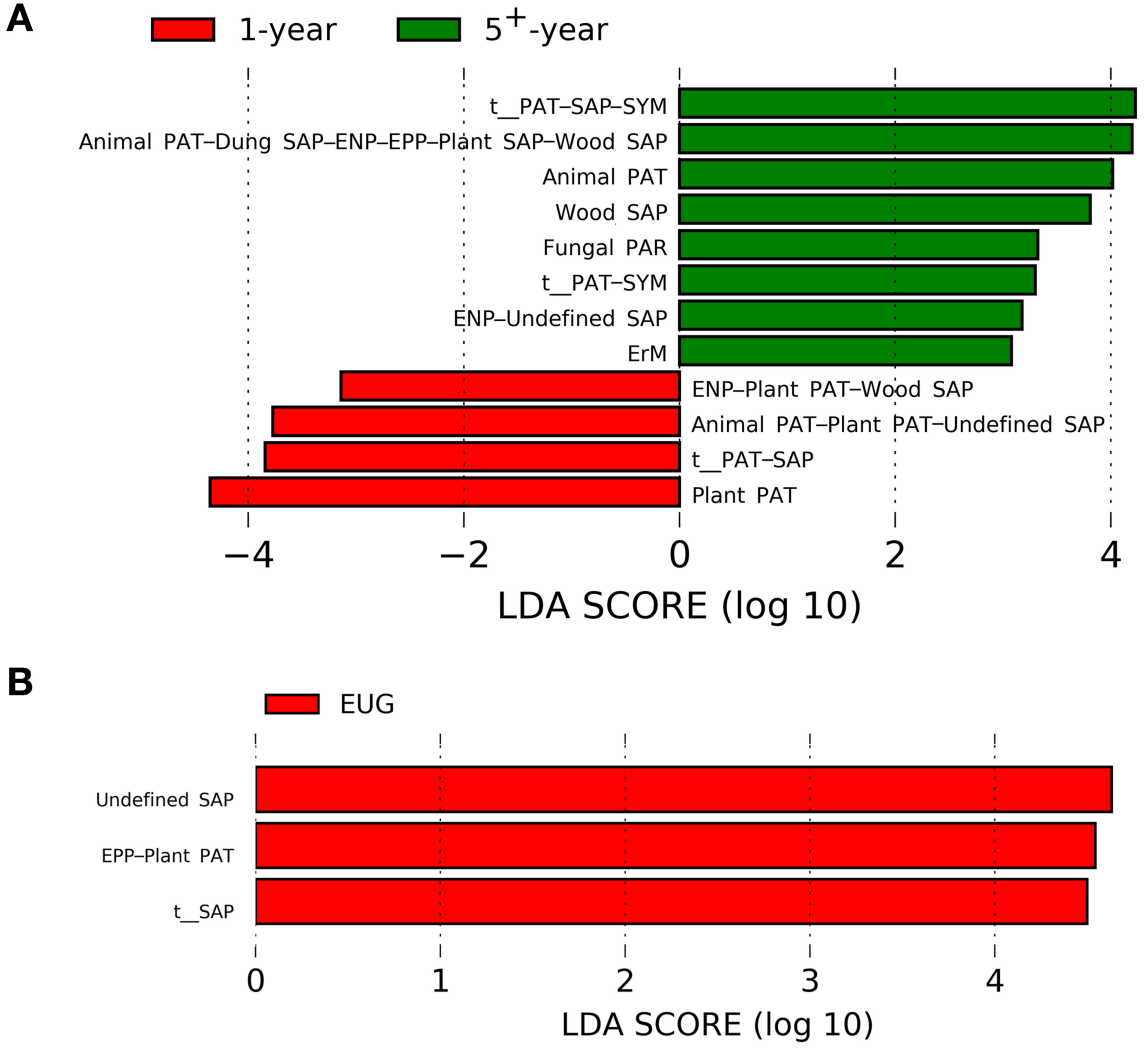
Fungal functional guilds significantly differentiated in *Eucalyptus* plantations with different **(A)** ages and **(B)** species identified by linear discriminant analysis coupled with effect size (LEfSe) using parameters of a threshold of LDA score > 3 and *p* < 0.05. EUG, *E. urophylla* × *E. grandis*; PAT, pathotroph; PAR, parasite; SAP, saprotroph; SYM, symbiotroph; ENP, endophyte; EPP, epiphyte; ErM, ericoid mycorrhizal.

For different plantation ages, the 1-year-old plantations had a higher abundance of plant pathogens, whereas the 5^+^-year-old plantations had a higher abundance of wood saprotrophs, endophytes–undefined saprotrophs, animal pathogens, fungal parasites, ericoid mycorrhizal fungi, and animal pathogens–dung, saprotrophs–endophytes–epiphytes–plant, and saprotrophs–wood saprotrophs (Figure 4A; *p* < 0.05). On the contrary, undefined saprotrophs and epiphytes–plant pathogens were more abundant in the EUG plantations (Figure 4B; *p* < 0.001). Out of the four dominant functional groups, plant pathogens were the most specialized group, followed by undefined saprotrophs, wood saprotrophs, and ECM (Supplementary Figure 5).

### Soil Physicochemical Properties in *Eucalyptus* Plantations

The physicochemical properties measured in the *Eucalyptus* plantations are summarized in Table 1. Among the sites, the mean SOC ranged from 2.96 to 4.06 g/kg, the TN content ranged from 0.089 to 0.266 g/kg, and the SWC ranged from 18.84 to 26.41%. The pH value in the EC5y-plantations was higher than those in the other sites (Table 1; *p* < 0.05). The C:N ratio, pH value, and the SOC content in the 5^+^-year-old plantations were higher than those in the 1-year-old plantations (Table 2; *p* < 0.05). On average, the pH value and the SWC were strongly affected by species (Table 2; *p* < 0.001). The interaction between plantation age and species had significant effects on the pH value, SWC, and TN (Table 2; *p* < 0.05).

**Table 1:** Soil physicochemical and microbial characteristics in *Eucalyptus* plantations.

**Factor**	**1year**			**5^+^year**		
	**EC1y**	**EUG1y**	**EP1y**	**EC5y**	**EUG5y**	**EP11y**
SOC (g/kg)	3.082 ± 0.193^c^	2.963 ± 0.182^c^	3.196 ± 0.177^bc^	3.854 ± 0.080^ab^	4.065 ± 0.148^a^	3.628 ± 0.012^abc^
TN (g/kg)	0.217 ± 0.020^ab^	0.182 ± 0.011^bc^	0.089 ± 0.003^d^	0.112 ± 0.012^d^	0.144 ± 0.026^bcd^	0.266 ± 0.014^a^
C:N ratio	13.486 ± 0.735^b^	17.474 ± 1.383^b^	37.974 ± 1.826^a^	36.954 ± 3.503^a^	33.345 ± 4.377^a^	12.725 ± 0.327^b^
pH	4.43 ± 0.04^c^	4.66 ± 0.02^b^	4.62 ± 0.06^bc^	5.31 ± 0.04^a^	4.68 ± 0.01^b^	4.37 ± 0.03^c^
SWC (%)	18.843 ± 0.712^c^	26.407 ± 0.332^a^	22.469 ± 0.973^ab^	19.792 ± 2.386^abc^	21.879 ± 0.847^ab^	24.717 ± 0.828^ab^
MB (g/m^2^)^R^	0.061 ± 0.010^a^	0.060 ± 0.013^a^	0.051 ± 0.011^a^	0.089 ± 0.013^a^	0.008 ± 0.001^b^	0.046 ± 0.007^a^
FB (10^5^ copy/g dry soil)^R^	300.593 ± 69.778^ab^	78.019 ± 28.854^bc^	8.876 ± 1.970^c^	1053.004 ± 228.429^a^	212.034 ± 44.532^ab^	266.163 ± 56.782^ab^
BB (10^6^ copy/g dry soil)^R^	106.653 ± 14.926^a^	32.964 ± 11.700^bc^	4.470 ± 0.839^c^	76.206 ± 14.600^ab^	78.966 ± 12.842^ab^	99.199 ± 18.675^ab^
FB:BB ratio^R^	0.273 ± 0.042^b^	0.262 ± 0.078^b^	0.208 ± 0.038^b^	1.344 ± 0.182^a^	0.269 ± 0.041^b^	0.262 ± 0.039^b^

*The values in the table are shown as the mean with standard error (n = 9). One-way ANOVA and Tukey honestly significant difference (HSD) post-hoc pairwise comparison tests (p < 0.05) were performed after checking for assumptions of normality and homoscedasticity. The letter after the value shows the significant difference in each measured property between plantations. Accessions with the same letter were not significantly different. The superscript ^R^ indicates that the soils at these sites had previously been characterized for microbial factors in Xu et al. (2020)*.

*EC, Eucalyptus camaldulensis; EP, Eucalyptus pellita; EUG, Eucalyptus urophylla × Eucalyptus grandis; 1y, 1 year; 5y, 5 years; 11y, 11 years; SOC, soil organic carbon; TN, total nitrogen; C:N ratio, ratio of soil organic carbon to total nitrogen; SWC, soil moisture content; MB, microbial biomass; FB, fungal biomass; BB, bacterial biomass; FB:BB ratio, ratio of fungal biomass to bacterial biomass*.

**Table 2:** Summary of two-way ANOVA model for responses of physicochemical and microbial characteristics to explanatory variables of *Eucalyptus* plantation age, species, and their interaction.

**Response variable**	**Explanatory variable**	**DF**	***F*** **-value**	***p*** **-value**
SOC	**Ages**	**1**	**42.28**	**<0.001**
	Species	2	0.3	0.75
	Interaction	2	2.69	0.08
TN	Ages	1	0.76	0.39
	Species	2	0.52	0.6
	**Interaction**	**2**	**42.42**	**<0.001**
C:N ratio	**Ages**	**1**	**5.32**	**0.03**
	Species	2	0.03	0.99
	**Interaction**	**2**	**55.21**	**<0.001**
pH	**Ages**	**1**	**45.26**	**<0.001**
	**Species**	**2**	**46.37**	**<0.001**
	**Interaction**	**2**	**111.6**	**<0.001**
SWC	Ages	1	0.33	0.57
	**Species**	**2**	**9.17**	**<0.001**
	**Interaction**	**2**	**3.23**	**0.047**
MB	Ages	1	1.46	0.23
	**Species**	**2**	**8.71**	**<0.001**
	**Interaction**	**2**	**7.99**	**0.001**
FB	**Ages**	**1**	**37.91**	**<0.001**
	**Species**	**2**	**19.48**	**<0.001**
	Interaction	2	3.12	0.053
BB	**Ages**	**1**	**11.19**	**0.002**
	**Species**	**2**	**5.23**	**0.009**
	**Interaction**	**2**	**10.99**	**<0.001**
FB:BB ratio	**Ages**	**1**	**29.16**	**<0.001**
	**Species**	**2**	**27.76**	**<0.001**
	**Interaction**	**2**	**24.59**	**<0.001**

*Explanatory variables deemed statistically significant are in bold*.

*SOC, soil organic carbon; TN, total nitrogen; C:N ratio, ratio of soil organic carbon to total nitrogen; SWC, soil moisture content; MB, microbial biomass; FB, fungal biomass; BB, bacterial biomass; FB:BB ratio, ratio of fungal biomass to bacterial biomass*.

The results of the Spearman correlation showed that the total nitrogen content of the soil was significantly negatively correlated with pH value (Supplementary Figure 6; pH vs. TN: *R* = −0.5758, *p* < 0.001), whereas the C:N ratio and FB:BB ratio showed an opposite correlation with pH (C:N vs. pH: *R* = 0.6527, *p* < 0.001; FB:BB vs. pH: *R* = 0.4504, *p* = 0.039). Linear regression analysis showed that the C:N ratio, pH value, SOC, and soil TN content were significantly correlated with *Eucalyptus* plantation age (Supplementary Figure 7; Adj. *R*^2^ >0.25, *p* < 0.01). Furthermore, fungal richness and some dominant fungal taxa and functional groups also showed significant correlations with various physicochemical and microbial factors. For instance, Eurotiales, *Curvularia, Coniosporium, Talaromyces*, and *Pisolithus* exhibited a significantly negative correlation with SOC (Supplementary Figure 8; *R* < −0.51, *p* < 0.05). In addition, plant pathogens and undefined saprotrophs exhibited a negative correlation with SOC (Supplementary Figure 9; *R* < -0.51, *p* < 0.05).

### Environmental Factors Contributing to Fungal Community and Potential Functional Structures

**Figure 5:**
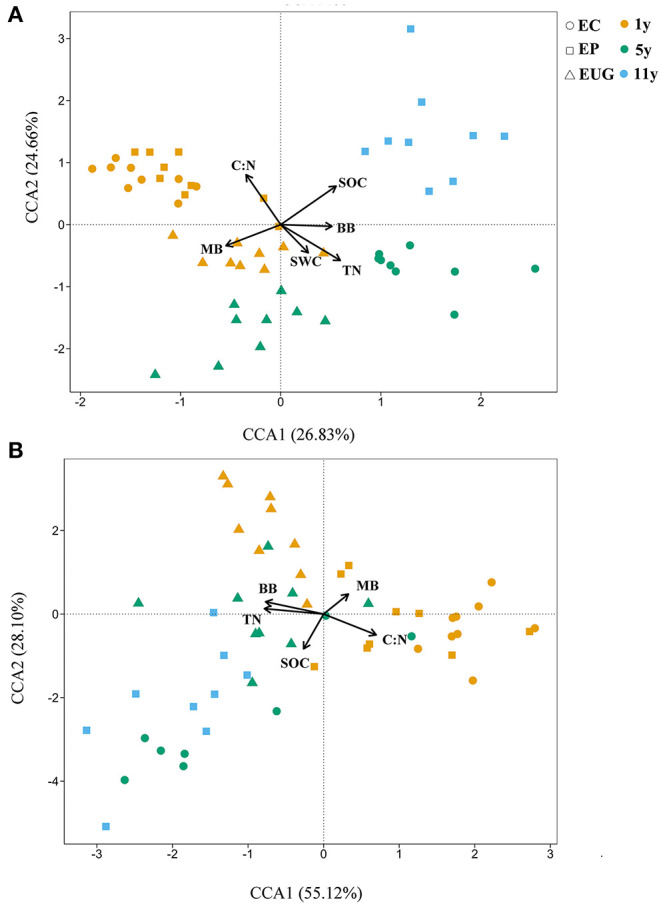
Canonical correspondence analyses (CCAs) showing **(A)** fungal and **(B)** functional community structures in *Eucalyptus* plantations with different ages and species with environmental variables as explanatory factors. EC, *E. camaldulensis*; EP, *E. pellita*; EUG, *E. urophylla* × *E. grandis*; 1y, 1 year; 5y, 5 years; 11y, 11 years; SOC, soil organic carbon; TN, total nitrogen; C:N ratio, ratio of soil organic carbon to total nitrogen; SWC, soil moisture content; MB, microbial biomass; BB, bacterial biomass.

The results of the canonical correspondence analyses showed that the physicochemical and microbial factors together explained 51.49% (CCA1: 26.83% and CCA2:24.66%) of the total variations in fungal community structure (Figure 5A). According to the correlation coefficients of the CCA, the C:N ratio, SOC, and TN content were the top three explanatory factors contributing to the variations in fungal community structures (Figure 5A; *R*^2^ >0.5, *p* < 0.001). In addition, the C:N ratio and MB were significantly correlated with fungal community structures in the 1-year-old plantations. The C:N ratio, BB, and TN content were the top three factors explaining the total variations in functional structures (83.22%) (Figure 5B; *R*^2^ >0.3, *p* < 0.001). Similar to the correlation coefficient of the fungal community structure, MB was significantly correlated with the functional community structures in the 1-year-old plantations.

## Discussion

In this study, we investigated the responses of fungal community and potential function to *Eucalyptus* plantation age and species. Plantation age seemed to have the largest contribution to the variations in fungal community structure compared with species, particularly for certain taxa of Basidiomycota. Plantation age, species, and their interaction also impacted the major soil physicochemical and microbial characteristics in the *Eucalyptus* plantations which, in turn, affected the fungal community. Previous studies have shown that the indirect influence between fungal community structure and soil pH is mediated by fungal and bacterial communities (Rousk et al., 2010), which is in line with the findings that the FB:BB ratio was positively correlated with fungal biomass (FB) and showed a negative correlation with pH (Supplementary Figure 6). Meanwhile, the abundance of several taxa of Ascomycota varied along with plantation age, and the abundance of Eurotiales and soil organic carbon (SOC) showed a negative correlation. Moreover, only *Pisolithus* and *Sebacina*, belonging to Basidiomycota, differentially changed in response to physicochemical and microbial characteristics. These data may indicate that, similar to poplar plantations (Zheng et al., 2017), Ascomycota is relatively more sensitive to changes in soil physicochemical properties in the *Eucalyptus* plantations.

Three of the most popular planted *Eucalyptus* species and two plantation ages were studied in this experiment. Especially for the species of *Eucalyptus pellita*, there was no 5-year-old plantation to compare with the other two species, which might limit the observations. In forest practice, it is easy to have mixed plantations of different *Eucalyptus* species. Mixed plantations of equal tree age are uncommon. The EP plantations of 1 and 11 years of age, however, showed more or less a similar trend with the other two species with 1- and 5-year-old plantations. We suggest that the results may still reflect the general effect of the age of the plantations. However, further investigations with more species and different plantation ages are needed to draw solid conclusions. In addition, a designated negative control, which could be unplanted soil next to the existing plantations, was lacking in this study and is necessary to validate the observations.

The age of the *Eucalyptus* plantations was an important driver of fungal alpha diversity and structure in this study. This is consistent with previous studies showing that changes in soil biodiversity in *Eucalyptus* plantations depend on the age of the plantation and are in parallel with the succession process of *Eucalyptus* trees (Chen et al., 2011; Zhang et al., 2013). Similar observation results were obtained in boreal *Pinus sylvestris* (Lim and Berbee, 2013) and *Tsuga mertensiana* (Kyaschenko et al., 2017) forests, northern temperate *Quercus mongolica* (He et al., 2016) and *Pinus koraiensis* (Zhiguang et al., 2016) forests, and Chinese subtropical *Phyllostachys praecox* (Li et al., 2017) forest, in which the changes in the fungal community were likely parallel with shifts in soil properties and vegetation composition caused by potential feedback on the tree stand age.

The age of *Eucalyptus* plantations affects fungal community richness, alpha diversity, and evenness in a different way. With the increase in the age of *Eucalyptus* plantations, the richness of the fungal communities increased, whereas the diversity and evenness decreased. Fungi are capable of degrading plant residues, although some of the litter inputs (e.g., lignin) were recalcitrant (Zheng et al., 2017). The higher fungal richness in older *Eucalyptus* plantations agreed with previous observations that the 5^+^-year-old plantations received more litter inputs with lower disturbance (e.g., fertilization and weeding) than the 1-year-old plantations (Chen et al., 2011). With increased plantation age, *Eucalyptus* root exudates contained more allelochemicals, which have inhibiting effects on the production and germination of fungal spores (Batish et al., 2008; Chen et al., 2011). This may cause changes in the abundance of certain fungal groups, resulting in a decrease in evenness and alpha diversity of fungal communities. This conclusion is also in line with the result that fungal diversity and evenness had a positive correlation.

With the increased age of *Eucalyptus* plantations, changes in fungal community structure were apparent at both low and high taxonomic levels. Several studies have shown that the abundance of Ascomycota decreased, while that of Basidiomycota increased in forest soil across plantation age (Zhiguang et al., 2016; Zheng et al., 2017). In this study, the relative abundance of phylum Ascomycota and its orders Pleosporales, Chaetothyriales, and Eurotiales decreased, whereas that of phylum Basidiomycota and its orders Agaricales, Sebacinales, Cantharellales, and Russulales increased with the increase in the age of the *Eucalyptus* plantations. The increased plant residues and components (e.g., lignin), with the increase in the age of the *Eucalyptus* plantations, might contribute to the observations. In the 1-year-old plantations, *Eucalyptus* trees were in a fast-growing stage with less plant residues containing little lignin after a clear-cutting of the vegetation cover (Cortez et al., 2014). Many fungal groups with cellulolytic ability within Ascomycota, including Pleosporales, Chaetothyriales, Eurotiales, *Talaromyces, Penicillium*, and *Aspergillus*, had a high abundance in 1-year-old plantations. These fungi are able to degrade cellulose in the early stages of decomposition (Ma et al., 2013; Galitskaya et al., 2017; Zheng et al., 2017; Zhao et al., 2018). Recalcitrant plant components became more common in the 5^+^-year-old plantations, where the orders Agaricales, Cantharellales, and Russulales, belonging to Basidiomycota, with the ability to degrade complex polymers (e.g., lignin) were expected to be more abundant (Žifcakova et al., 2011; Nagy et al., 2015). Unlike most of the taxa in Ascomycota, the order Helotiales, which was described as saprotrophs in this study, was enriched in the 5^+^-year-old plantations similar to Basidiomycota. Previous studies have shown that Helotiales fungi can effectively degrade the allelochemicals produced by *Eucalyptus*, whereas the activities of members of *Aspergillus, Penicillium*, and *Trichoderma*, which were also described as saprotrophs, were strongly inhibited by *Eucalyptus* essential oil (Batish et al., 2008; Liu et al., 2018).

Previous studies have shown that plantation age affects vegetation biomass and plant residue composition and indirectly changes the physical and chemical properties of soil (Zhiguang et al., 2016; Zheng et al., 2017). The SOC was the most important factor influencing the fungal community structure across stand age in a Chinese subtropical forest (Wu et al., 2013). In this study, most of the core OTUs were described as saprotrophs, and the SOCs were identified as one of the key drivers in the fungal community and functional structures. Members of Pleosporales, Chaetothyriales, and Eurotiales (Ascomycota) have been described previously as saprotrophs and play a crucial role in soil organic matter degradation, and their relative abundance had a significantly positive correlation with SOC mineralization rate (Zheng et al., 2017). In this study, the orders Pleosporales, Chaetothyriales, and Eurotiales (Ascomycota) decreased in abundance along with plantation age, and Eurotiales and its genus *Talaromyces, Curvularia* (Pleosporales), and *Coniosporium* (Chaetothyriales) exhibited a significantly negative correlation with the SOC level. These results support previous observations regarding the role of SOC on fungal community structure. In addition, the organic matter derived from fungi is biochemically more resistant and can enhance C storage and slow the turnover of soil organic matter depending on the fungal community (Bailey et al., 2002; Zheng et al., 2017). This could be another association between SOC and fungal community structure.

By FUNGuild analysis of potential function, both saprotrophs and pathotrophs shifted in abundance between plantation age, in which wood saprotrophs, dung saprotrophs, fungal parasites, and animal pathogens increased in abundance, whereas plant pathogens decreased in abundance along with an increase in plantation age. This shift was likely related to the species richness and diversity of epiphytes, bryophytes, and soil animals, which were positively correlated with plantation age because of the presence of numerous microhabitats and habitat continuity in the older forests (Kantvilas and Jarman, 2004; Paillet et al., 2010; Chen et al., 2011; Davey et al., 2014). In addition, the taxa of plant pathogens tended to be relatively more abundant in 1-year-old *Eucalyptus* plantations. This result is inconsistent with the observation results obtained from *Xanthoceras sorbifolia* and *Populus* spp. plantations, where the abundance of certain plant pathogens increased across stand age (Jing et al., 2011; Zhu et al., 2015). This might be due to *Eucalyptus* essential oils and their major constituent that possess antifungal activity against a wide range of pathogenic fungi, including several species of *Aspergillus* and *Penicillium* (Nakahara et al., 2003; Kaur et al., 2012), which had a lower abundance in 5^+^-year-old *Eucalyptus* plantations. Some species of *Mortierella*, which were mainly composed of Mortierellomycota, can produce potential antagonistic compounds against various plant pathogens (Tagawa et al., 2010; Qiao et al., 2017). Therefore, soils with a higher abundance of *Mortierella* might receive less soil-borne diseases (Qiao et al., 2017). Notably, it is important to bear in mind that the FUNGuild analysis performed in this study was based on species classification, which can only predict potential rather than true fungal functions. Due to such limitation, a more comprehensive functional analysis using methods, such as metagenomics or real-time quantitative PCR, is necessary to further elucidate and test the community functional structure.

In this study, the *Eucalyptus* species had no effects on fungal alpha diversity. This result partially supports the findings that tree species do not result in changes in fungal alpha diversity (Davey et al., 2014). The fungal communities included saprotrophs, symbiotrophs, and pathotrophs. Changes in tree species may cause a shift in the abundance of certain fungal groups. It is likely that fungal species with similar functions could replace others, resulting in no net change in alpha diversity and function. Although *Eucalyptus* species partially explained the variation in fungal community structure, only a small number of biomarkers and functional groups differed significantly among the different *Eucalyptus* species. For instance, both of the order Agaricales and its genus *Coprinellus*, and undefined saprotrophs, which had higher host specificity, were more abundant in the EUG plantation. This proves the significant correlation between fungal community structure and predicted functional community structure, because most of the members of Agaricales and *Coprinellus* were described as undefined saprotrophs in this study. Meanwhile, a recent study showed similar results that plant pathogens and saprotrophs can better highlight host preference and/or specificity than ECM fungi (Chen et al., 2019). Different tree species can influence soil microbial biomass, fungal community, and functional structure through the quantity and quality of plant residues (Zeng et al., 2015). The soil microbial biomass (MB), fungal biomass (FB), and bacterial biomass (BB) were strongly affected by *Eucalyptus* species (Xu et al., 2020). Interestingly, the SOC in this study was also affected by the *Eucalyptus* species, which showed significant correlations with the fungal community in the EC5- and EP11y-plantations, but a negative correlation in the EUG5y-plantation. The leaf traits were similar between closely related *Eucalyptus* species and differed among distantly related species (Jie et al., 2018). Molecular evidence showed that *E. urophylla* × *E. grandis* (EUG) and *E. pellita* (EP) had higher genetic similarities than *E. camaldulensis* (EC) (Zeng et al., 2010). The fungal community and functional structures in the plantations of *E. pellita* and *E. camaldulensis* were more similar than those of *E. urophylla* × *E. grandis*; especially, the EP1y- and EC1y-plantations were not significantly different by the fungal community. One explanation for this observed discrepancy might be that *Eucalyptus* root exudates significantly affect the soil fungal community structure besides the effects of SOC (Wu and Yu, 2019). A previous study has demonstrated that the root exudates of *E. grandis* (male parent of *E. urophylla* × *E. grandis*) can significantly stimulate the growth of members of *Pisolithus* (Lagrange et al., 2001). In this study, *P. orientalis* was more abundant in the EUG plantations. Therefore, how *Eucalyptus* root exudates affect the soil fungal community and the influence of *Eucalyptus* species on the compositions of root exudates need to be further investigated.

## Conclusions

In brief, both the age of the *Eucalyptus* plantations and species contributed to the shifts in fungal community and functional structures, in which the age of the plantations had stronger effects than the *Eucalyptus* species. The age of the *Eucalyptus* plantations, rather than species, affected the fungal alpha community diversity. With the increase in the age of the plantations, the abundance of Ascomycota decreased, while that of Basidiomycota increased. The predicted fungal community function shifted from the dominance of plant pathogens to the abundance of saprotrophs and symbiotrophs. The SOC was the key driver contributing to the shift in the fungal community structure and potential function. The results highlight the importance of the age of *Eucalyptus* plantations and species on the fungal community structure under atropical monsoon climate.

## Data Availability Statement

The datasets presented in this study can be found in online repositories. The names of the repository/repositories and accession number(s) can be found at: https://www.ncbi.nlm.nih.gov/, PRJNA580298.

## Author Contributions

HS: conceptualization, investigation, project administration, and funding acquisition. HS and BL: methodology, software, and formal analysis. ZQ, YM, and JX: validation, resources, and supervision. BL: data curation, writing—original draft preparation, and visualization. HS and PC: writing—review and editing. All the authors have read and agreed to the published version of the manuscript.

## Funding

This research was funded by the National Natural Science Foundation of China (31870474), research funding for the Jiangsu Specially Appointed Professor (project 165010015), research funding for the Postgraduate Research & Practice Innovation Program of Jiangsu Province (KYCX19_1076), and research funding for the Priority Academic Program Development (PAPD) of Jiangsu Higher Education Institutions.

## Conflict of Interest

The authors declare that the research was conducted in the absence of any commercial or financial relationships that could be construed as a potential conflict of interest.

## Publisher's Note

All claims expressed in this article are solely those of the authors and do not necessarily represent those of their affiliated organizations, or those of the publisher, the editors and the reviewers. Any product that may be evaluated in this article, or claim that may be made by its manufacturer, is not guaranteed or endorsed by the publisher.
