# Supplementary material for: Eucalyptus Plantation Age and Species Govern Soil Fungal Community Structure and Function Under a Tropical Monsoon Climate in China
Source: Front Fungal Biol. 2021 Sep 10;2:703467. doi: 10.3389/ffunb.2021.703467 (PMC10512287; doi:10.3389/ffunb.2021.703467)
Supplement: Supplementary file 1 [file Data_Sheet_1.pdf]

Supplementary Tables

Table S1. Spearman correlation analyses were performed between fungal community richness (Chao 1), diversity (Invsimpson), and evenness (Simpsoneven). Spearman correlation deemed statistically significant are bolded.

Factor	R value	P value
Chao 1 vs Invsimpson	0.2066	0.5358
Chao 1 vs Simpsoneven	-0.0127	1
**Invsimpson vs Simpsoneven**	**0.9184**	**<0.001**

Supplementary Figures

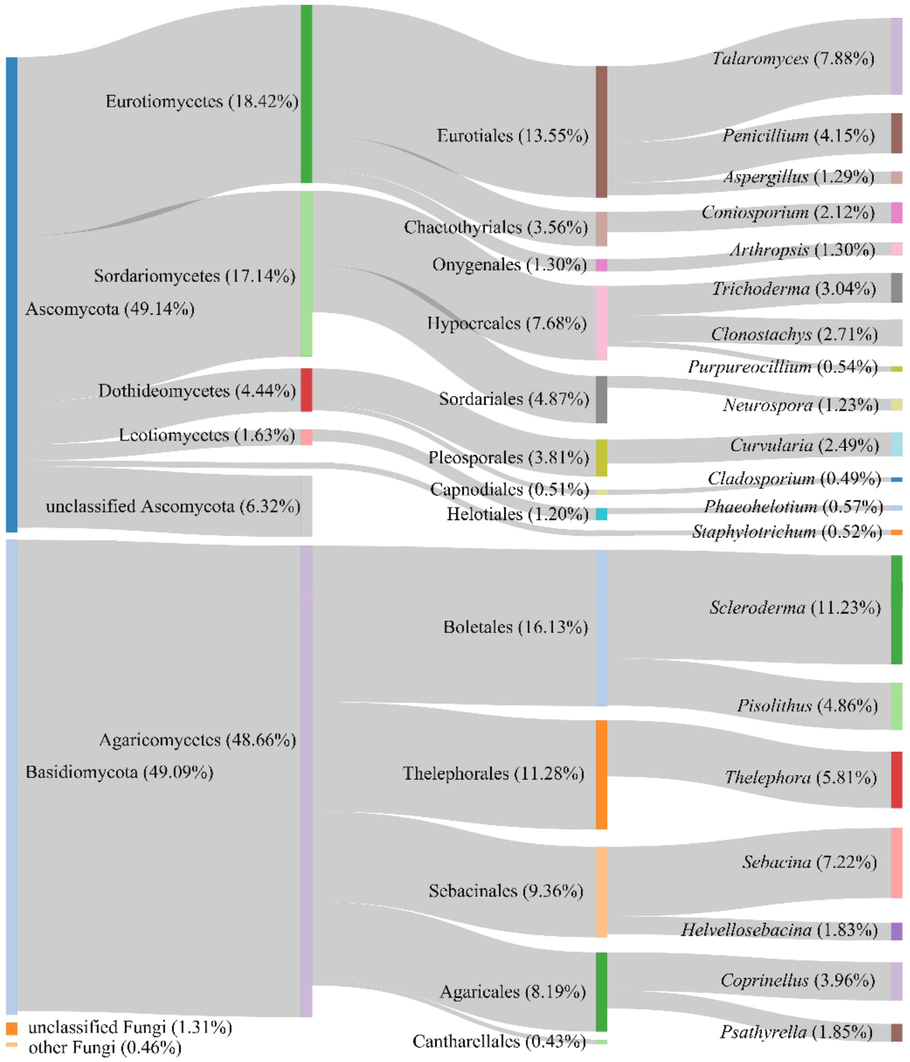

Figure S1. Sankey chart showing the overall distribution of fungal sequences (n = 1,791,002) at the phylum, class, order, and genus (top20) levels.

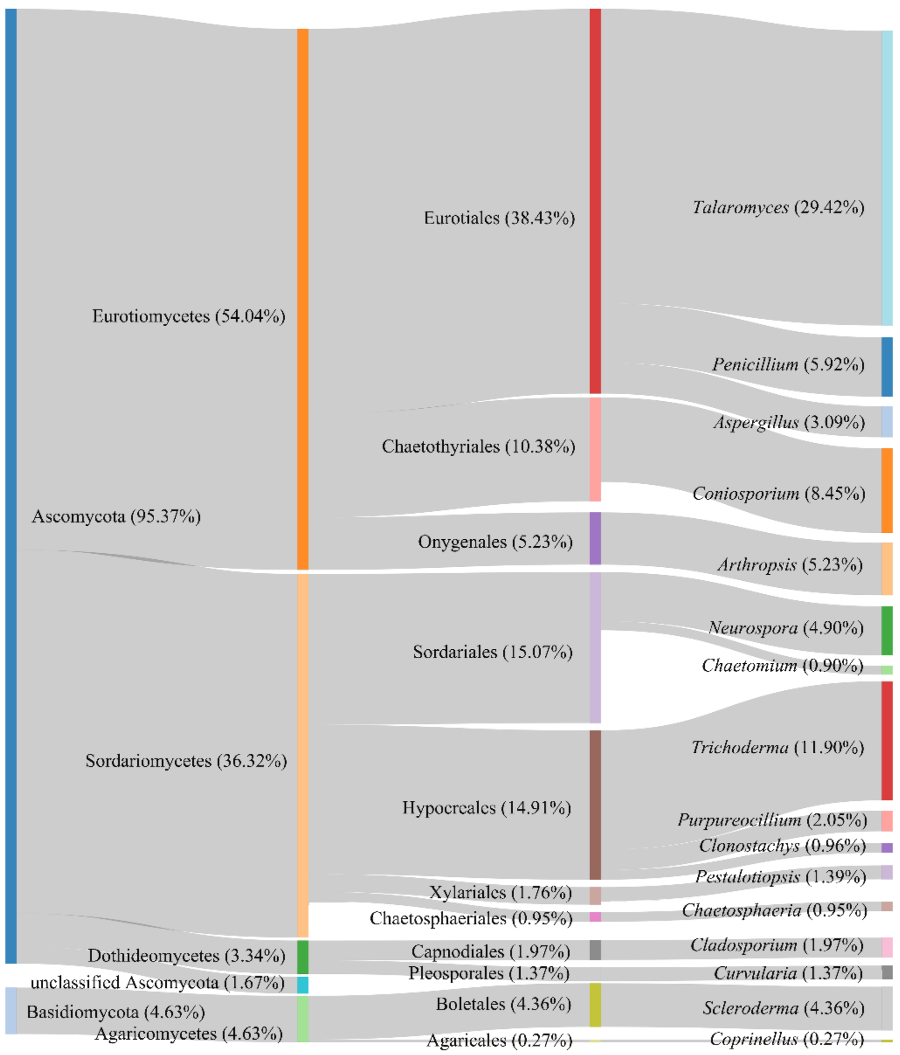

Figure S2. Sankey chart showing core OTUs (sequences n = 444,849) existed in more than half of the soils from the *Eucalyptus* plantations and had average relative abundances > 0.1% in at least one plantation

A

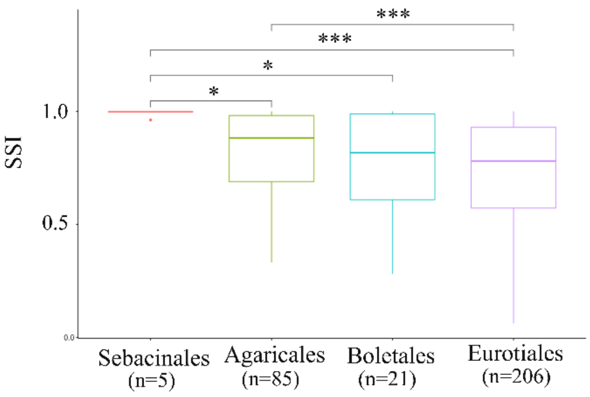

B

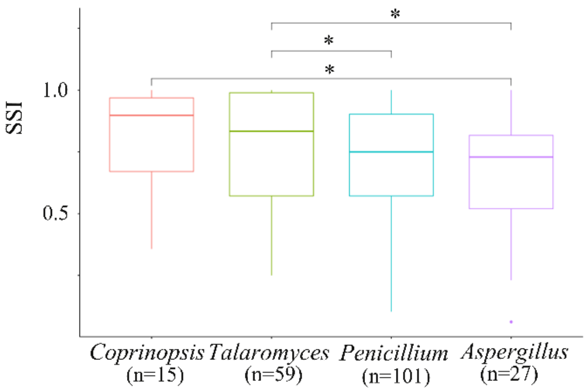

Figure S3. Box plots showing the distribution of the Species Specialization Index (SSI) for the dominant fungal order (**A**) and genus (**B**). SSI differences are significantly different for all pairwise by two-tailed Mann-Whitney U test. The significances of correlation analyses are marked with asterisks (*) at different significance levels (* for $P < 0.05$, ** for $P < 0.01$ and *** for $P < 0.001$).

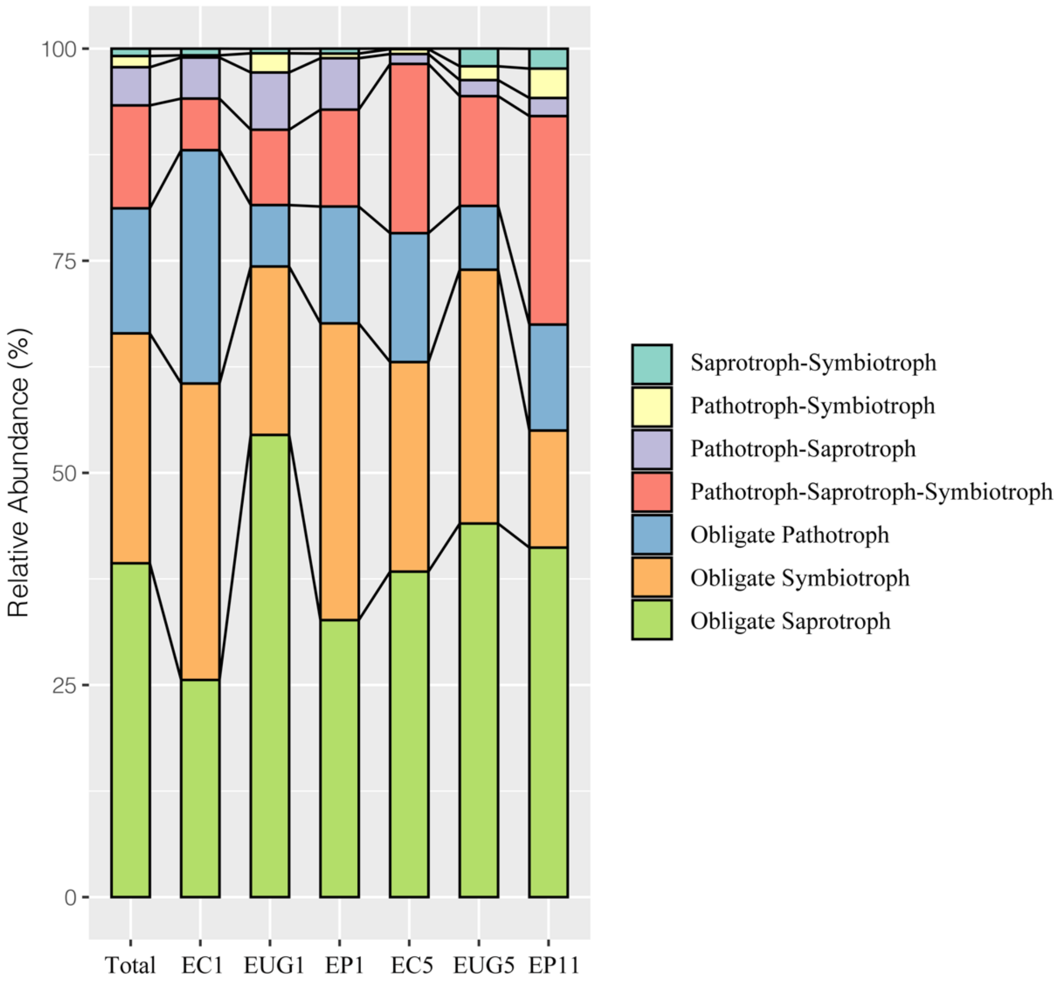

Figure S4. Sankey chart showing the overall distribution of the functional guilds (*n* = 1,060,621). Abbreviation: EC: *E. camaldulensis*; EP: *E. pellita*; EUG: *E. urophylla*×*E. grandis*; 1y: 1 year; 5y: 5 years; 11y: 11 years.

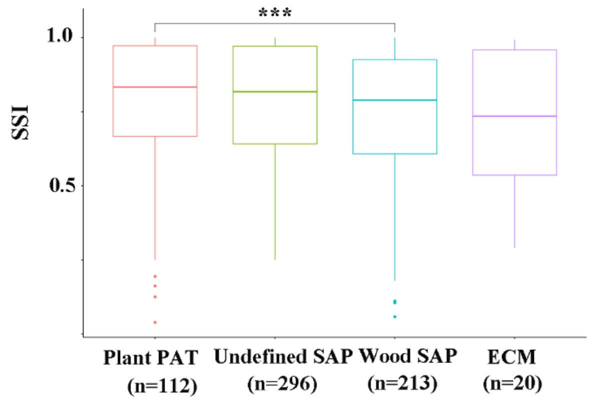

Figure S5. Box plots showing the distribution of the Species Specialization Index (SSI) for the dominant functional guilds. SSI differences are significantly different for all pairwise by two-tailed Mann-Whitney U test. The significances of correlation analyses are marked with asterisks (*) at different significance levels (* for $P < 0.05$, ** for $P < 0.01$ and *** for $P < 0.001$).

Abbreviation: PAT: pathotroph; SAP: saprotroph; ECM: Ectomycorrhizal.

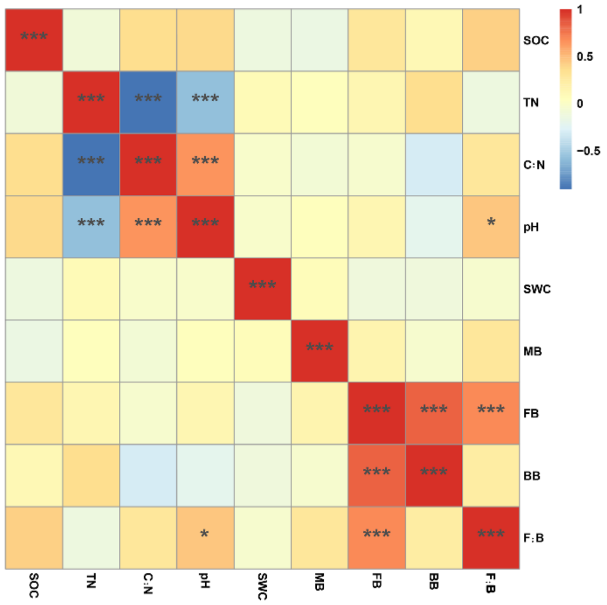

Figure S6. Spearman correlation analyses were performed between physico-chemical and microbial characteristics. The red and blue colors indicate positive and negative correlations, respectively. The significances of correlation analyses are marked with asterisks (*) at different significance levels (* for $P < 0.05$, ** for $P < 0.01$ and *** for $P < 0.001$).

Abbreviation: SOC: soil organic carbon; TN: total nitrogen; C:N ratio: ratio of soil organic carbon to total nitrogen; SWC: soil moisture content; MB: microbial biomass; FB: fungal biomass; BB: bacterial biomass; F:B ratio: ratio of fungal biomass to bacterial biomass.

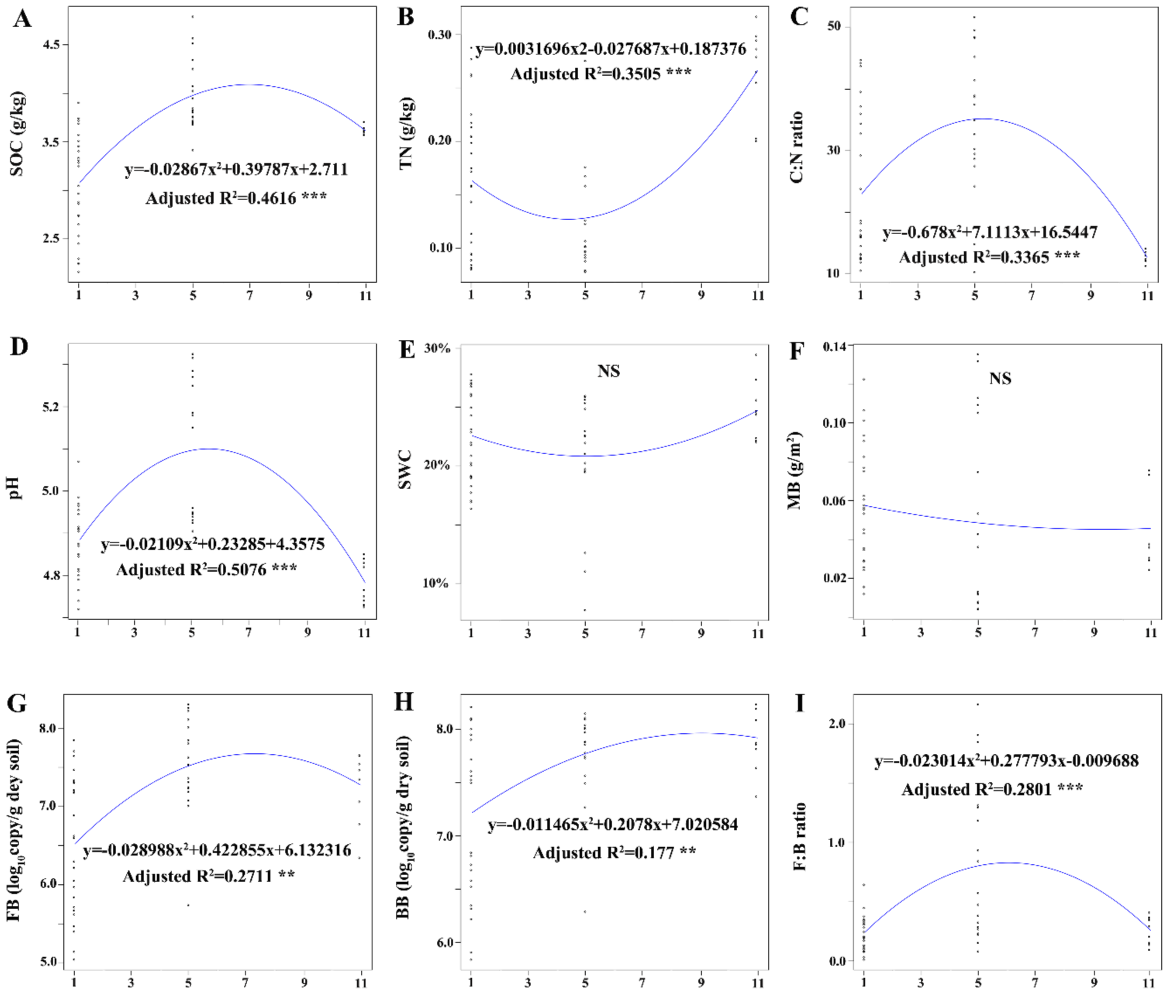

Figure S7. The trend of physico-chemical and microbial characteristics related to the *Eucalyptus* plantation age using linear regression analysis. The significances of correlation analyses are marked with asterisks (*) at different significance levels (* for $P < 0.05$, ** for $P < 0.01$ and *** for $P < 0.001$).

Abbreviation: SOC: soil organic carbon; TN: total nitrogen; C:N ratio: ratio of soil organic carbon to total nitrogen; SWC: soil moisture content; MB: microbial biomass; FB: fungal biomass; BB: bacterial biomass; F:B ratio: ratio of fungal biomass to bacterial biomass.

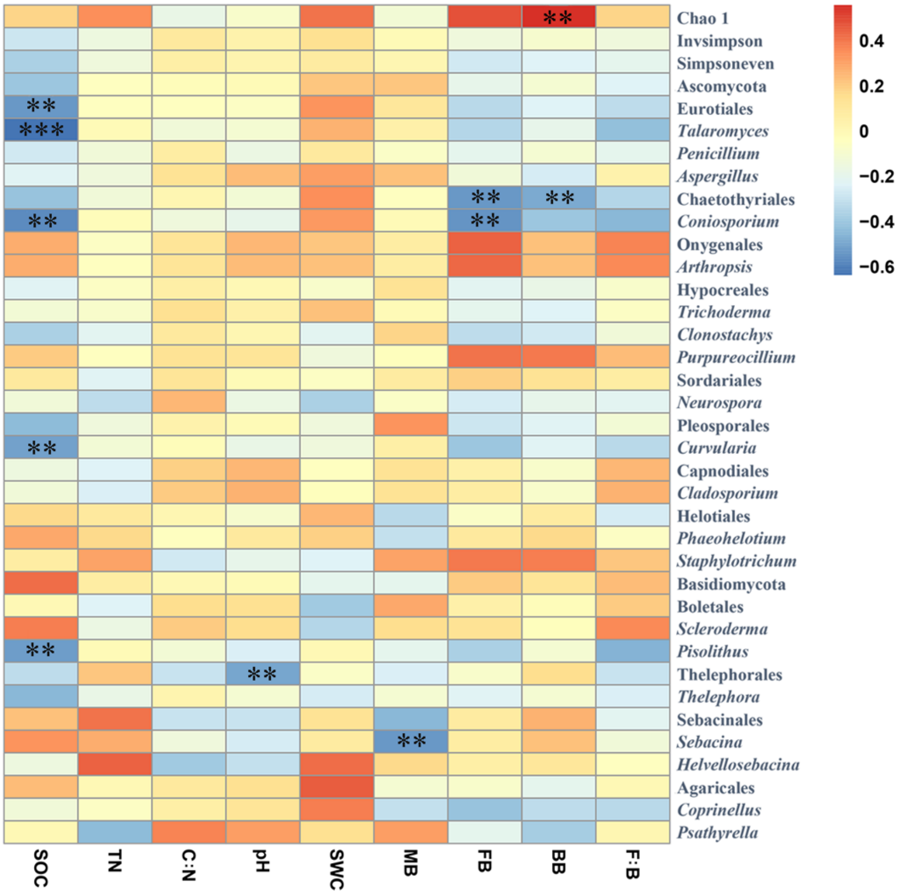

Figure S8. Spearman correlation analyses were performed between dominant fungal taxa and physico-chemical and microbial characteristics. The red and blue colors indicate positive and negative correlations, respectively. The significances of correlation analyses are marked with asterisks (*) at different significance levels (* for $P < 0.05$, ** for $P < 0.01$ and *** for $P < 0.001$).

Abbreviation: SOC: soil organic carbon; TN: total nitrogen; C:N ratio: ratio of soil organic carbon to total nitrogen; SWC: soil moisture content; MB: microbial biomass; FB: fungal biomass; BB: bacterial biomass; F:B ratio: ratio of fungal biomass to bacterial biomass.

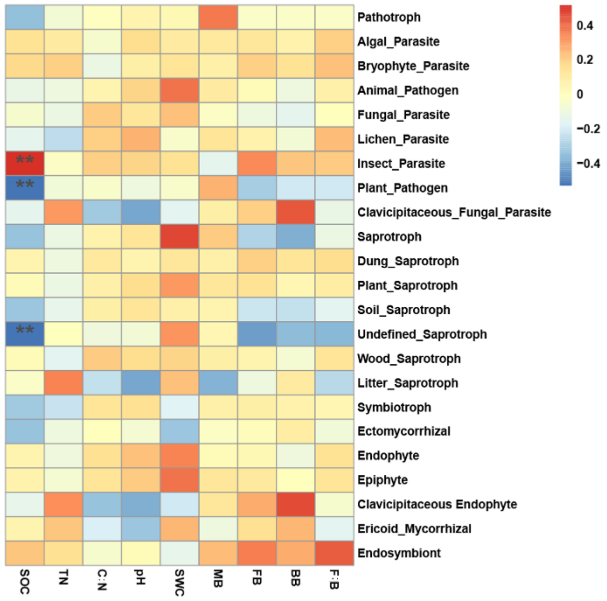

Figure S9. Spearman correlation analyses were performed between functional guilds and physicochemical and microbial characteristics. The red and blue colors indicate positive and negative correlations, respectively. The significances of correlation analyses are marked with asterisks (*) at different significance levels (* for $P < 0.05$, ** for $P < 0.01$ and *** for $P < 0.001$)

Abbreviation: SOC: soil organic carbon; TN: total nitrogen; C:N ratio: ratio of soil organic carbon to total nitrogen; SWC: soil moisture content; MB: microbial biomass; FB: fungal biomass; BB: bacterial biomass; FB:BB ratio: ratio of fungal biomass to bacterial biomass.
